# Editorial for the Special Issue “Chronic Neuropathic Pain Therapy and Anaesthesia”

**DOI:** 10.3390/medicina59040674

**Published:** 2023-03-29

**Authors:** Grzegorz Miękisiak

**Affiliations:** Institute of Medicine, University of Opole, 45-040 Opole, Poland; gmiekisiak@gmail.com

Chronic neuropathic pain (CNP), a complex and debilitating condition arising from damage or dysfunction of the somatosensory nervous system, affects millions of people worldwide [1]. Traditional pharmacologic and non-pharmacologic treatments have provided limited relief for many patients, necessitating the development of novel therapies to improve outcomes [2]. New trends in the treatment of neuropathic pain are focused on innovative approaches that go beyond traditional pharmacological therapies. These trends aim to provide more effective and individually tailored treatment options for patients with CNP.

The growing field of personalized medicine and pharmacogenomics is transforming the way we approach CNP management. By identifying specific genetic variations that influence an individual’s response to analgesic medications [3,4], healthcare providers can tailor treatment strategies to optimize efficacy and minimize adverse effects. Additionally, understanding the genetic predisposition to neuropathic pain may facilitate early intervention and prevention strategies. At least 28 genes have been identified which are significantly associated with this pathology, mainly involved in neurotransmission, immunologic response, and metabolism [5]. A rapidly growing knowledge base on molecular mechanisms has allowed for the introduction of gene therapy, which is a relatively new but promising field. In the context of neuropathic pain, gene therapy seeks to target the underlying molecular and cellular mechanisms that contribute to the development and maintenance of pain. Some potential gene therapy approaches for neuropathic pain include the modulation [6] or silencing [7] of pain-related genes or targeting ion channels [8]. Another promising avenue is the use of gene therapy in combination with regenerative medicine approaches, such as stem cell therapy, to promote the repair and regeneration of damaged nervous tissue [9,10]. By introducing genes that promote nerve growth, reduce inflammation, and modulate immune responses, gene therapy may enhance the effectiveness of regenerative medicine in treating CNP.

For years, neuromodulation has been a vital component in treating neuropathic pain, and recent advancements have further enhanced its effectiveness. In recent years, emerging neuromodulation techniques have been developed to offer additional treatment options. High-Frequency Spinal Cord Stimulation (HF-SCS) utilizes significantly higher frequencies, up to 10,000 Hz, compared to the traditional stimulation approach, which operates within a 40 to 60 Hz frequency range. HF-SCS has been shown to provide better pain relief and fewer side effects, such as paresthesia, compared to traditional SCS [11]. However, frequency is not the sole parameter that has been enhanced to achieve greater efficacy. Burst Spinal Cord Stimulation (SCS) provides stimulation in brief, high-frequency bursts instead of continuous pulses. It has demonstrated promising results in both clinical and laboratory environments, indicating its potential for future applications [12]. However, the spinal cord is not the only target for stimulation when it comes to treating neuropathic pain. Dorsal root ganglion stimulation (DRG-S) targets the dorsal root ganglion, a cluster of nerve cell bodies in the spinal nerves, which plays a crucial role in transmitting pain signals. DRG-S has been found to be effective in treating focal neuropathic pain conditions that are not adequately managed by traditional SCS [13].

On the other hand the peripheral nerve stimulation (PNS) involves the placement of electrodes near peripheral nerves to modulate their activity. PNS is less invasive than SCS or DBS and has been shown to be effective in treating various neuropathic pain conditions, including post-surgical neuropathic pain and post-amputation pain [14]. However, recent years have also seen a surge in interest regarding non-invasive stimulation techniques for the treatment of neuropathic pain. Transcranial magnetic stimulation (TMS) is a non-invasive neuromodulation technique. It uses magnetic fields to stimulate the brain’s pain-processing regions. TMS has demonstrated efficacy in treating various types of chronic pain, including CNP [15]. Transcranial direct current stimulation (tDCS) is another non-invasive neuromodulation technique that uses weak electrical currents to modulate brain activity. Some studies have shown that tDCS can provide pain relief in patients with CNP [16], although further research is needed to establish its effectiveness and optimal parameters.

However, in the field of CNP pain treatment, researchers and clinicians are currently investigating a range of less conventional but promising approaches. As research continues to evolve, these rising therapies may eventually become more widely accepted and integrated into standard treatment protocols. Optogenetics is one of them. This technique involves the genetic modification of neurons to make them sensitive to light. By selectively activating or inhibiting specific neurons using light, optogenetics has the potential to provide precise and targeted neuromodulation for the treatment of CNP [17]. The use of regenerative medicine, including stem cell therapy [18] and platelet-rich plasma injections [19], is also gaining popularity. These therapies aim to repair damaged nervous tissue, reduce inflammation, and promote healing, providing a novel approach to treating the underlying cause of CNP rather than just managing its symptoms.

Other alternative, innovative, and promising therapeutic pathways being explored include virtual reality (VR) and digital therapeutics. VR has been shown to be effective in reducing pain perception and improving the emotional well-being of patients after spinal cord injury [20], while digital therapeutics, including smartphone apps and online platforms, enable self-management and offer remote access to healthcare professionals for the treatment of chronic pain, regardless of its cause [21].

Without doubt the complexity of neuropathic pain necessitates a comprehensive and interdisciplinary approach to treatment. Incorporating various pharmacological, psychological, and physical therapies, multimodal pain management aims to address the multiple dimensions of pain, including sensory, emotional, and cognitive aspects [22,23]. Collaborative care involving physicians, nurses, pharmacists, physical therapists, and psychologists is crucial in ensuring a well-rounded, patient-centered treatment plan.

The future of CNP management is promising, with ongoing research and development of novel treatment strategies. Personalized medicine, multimodal and interdisciplinary pain management, neuromodulation therapies, regenerative medicine, and digital therapeutics are transforming the way we approach this challenging condition. Embracing these advances will be crucial in improving the quality of life for millions of individuals suffering from CNP.

## References

[1] Okerman T., Jurgenson T., Moore M., Klein A.H. (2021). Inhibition of the phosphoinositide 3-kinase-AKT-cyclic GMP-c-Jun N-terminal kinase signaling pathway attenuates the development of morphine tolerance in a mouse model of neuropathic pain. Mol. Pain.

[2] Bouhassira D., Attal N. (2019). The multiple challenges of neuropathic pain. Neurosci. Lett..

[3] Bates J., Fudin J., Patel J.N. (2022). Integrating pharmacogenomics into precision pain management. Support. Care Cancer.

[4] Yamamoto P.A., Costa A.C.C., Lauretti G.R., de Moraes N.V. (2019). Pharmacogenomics in chronic pain therapy: From disease to treatment and challenges for clinical practice. Pharmacogenomics.

[5] Veluchamy A., Hébert H.L., Meng W., Palmer C.N., Smith B.H. (2018). Systematic review and meta-analysis of genetic risk factors for neuropathic pain. Pain.

[6] Vallejo R., Kelley C.A., Gupta A., Smith W.J., Vallejo A., Cedeño D.L. (2020). Modulation of neuroglial interactions using differential target multiplexed spinal cord stimulation in an animal model of neuropathic pain. Mol. Pain.

[7] Mo K., Wu S., Gu X., Xiong M., Cai W., Atianjoh F.E., Jobe E.E., Zhao X., Tu W.-F., Tao Y.-X. (2018). MBD1 Contributes to the Genesis of Acute Pain and Neuropathic Pain by Epigenetic Silencing of *Oprm1* and *Kcna2* Genes in Primary Sensory Neurons. J. Neurosci..

[8] Stevens E.B., Stephens G.J. (2018). Recent advances in targeting ion channels to treat chronic pain. Br. J. Pharmacol..

[9] Manion J., Khuong T., Harney D., Littleboy J.B., Ruan T., Loo L., Costigan M., Larance M., Caron L., Neely G.G. (2020). Human induced pluripotent stem cell-derived GABAergic interneuron transplants attenuate neuropathic pain. Pain.

[10] Sarveazad A., Janzadeh A., Taheripak G., Dameni S., Yousefifard M., Nasirinezhad F. (2019). Co-Administration of Human Adi-pose-Derived Stem Cells and Low-Level Laser to Alleviate Neuropathic Pain after Experimental Spinal Cord Injury. Stem Cell Res. Ther..

[11] Dones I., Levi V. (2018). Spinal Cord Stimulation for Neuropathic Pain: Current Trends and Future Applications. Brain Sci..

[12] Chakravarthy K., Fishman M.A., Zuidema X., Hunter C.W., Levy R. (2019). Mechanism of Action in Burst Spinal Cord Stimulation: Review and Recent Advances. Pain Med..

[13] D’Souza R.S., Kubrova E., Her Y.F., Barman R.A., Smith B.J., Alvarez G.M., West T.E., Abd-Elsayed A. (2022). Dorsal Root Ganglion Stimulation for Lower Extremity Neuropathic Pain Syndromes: An Evidence-Based Literature Review. Adv. Ther..

[14] Char S., Jin M.Y., Francio V.T., Hussain N., Wang E.J., Morsi M., Orhurhu V., Prokop L.J., Fink A., D’Souza R.S. (2022). Implantable Peripheral Nerve Stimulation for Peripheral Neuropathic Pain: A Systematic Review of Prospective Studies. Biomedicines.

[15] Gatzinsky K., Bergh C., Liljegren A., Silander H., Samuelsson J., Svanberg T., Samuelsson O. (2020). Repetitive transcranial magnetic stimulation of the primary motor cortex in management of chronic neuropathic pain: A systematic review. Scand. J. Pain.

[16] Yang S., Chang M.C. (2021). Transcranial Direct Current Stimulation for the Management of Neuropathic Pain: A Narrative Review. Pain Physician.

[17] Guida F., De Gregorio D., Palazzo E., Ricciardi F., Boccella S., Belardo C., Iannotta M., Infantino R., Formato F., Marabese I. (2020). Behavioral, Biochemical and Electrophysiological Changes in Spared Nerve Injury Model of Neuropathic Pain. Int. J. Mol. Sci..

[18] Joshi H.P., Jo H.-J., Kim Y.-H., An S.-B., Park C.-K., Han I. (2021). Stem Cell Therapy for Modulating Neuroinflammation in Neuropathic Pain. Int. J. Mol. Sci..

[19] Bohren Y., Timbolschi D.I., Muller A., Barrot M., Yalcin I., Salvat E. (2022). Platelet-rich plasma and cytokines in neuropathic pain: A narrative review and a clinical perspective. Eur. J. Pain.

[20] Putrino D., Tabacof L., Breyman E., Revis J., Soomro Z., Chopra D., Delaney K., Smeragliuolo A., Cortes M. (2021). Pain Reduction after Short Exposure to Virtual Reality Environments in People with Spinal Cord Injury. Int. J. Environ. Res. Public Health.

[21] Eccleston C., Blyth F.M., Dear B.F., Fisher E.A., Keefe F.J., Lynch M.E., Palermo T.M., Reid M.C., de C Williams A.C. (2020). Managing patients with chronic pain during the COVID-19 outbreak: Considerations for the rapid introduction of remotely supported (eHealth) pain management services. Pain.

[22] Bahm J., Winkel R., Zyluk A. (2018). Neuropathic pain: We need more interdisciplinary and holistic treatment. Neurol. Psychiatry Brain Res..

[23] Varshney V., Osborn J., Chaturvedi R., Shah V., Chakravarthy K. (2021). Advances in the interventional management of neuropathic pain. Ann. Transl. Med..

